# A Non-Humanoid Robot in the “Uncanny Valley”: Experimental Analysis of the Reaction to Behavioral Contingency in 2–3 Year Old Children

**DOI:** 10.1371/journal.pone.0006974

**Published:** 2009-09-16

**Authors:** Kentaro Yamamoto, Saori Tanaka, Hiromi Kobayashi, Hideki Kozima, Kazuhide Hashiya

**Affiliations:** 1 Graduate School of Human-Environment Studies, Kyushu University, Fukuoka-shi, Fukuoka, Japan; 2 School of Education, Kyushu University, Fukuoka-shi, Fukuoka, Japan; 3 Faculty of Human-Environment Studies, Kyushu University, Fukuoka-shi, Fukuoka, Japan; 4 Department of Spatial Design and Information Systems, School of Project Design, Miyagi University, Kurokawa-gun, Miyagi; L'université Pierre et Marie Curie, France

## Abstract

Infants' sensitivity to social or behavioral contingency has been examined in the field of developmental psychology and behavioral sciences, mainly using a double video paradigm or a still face paradigm. These studies have shown that infants distinguish other individuals' contingent behaviors from non-contingent ones. The present experiment systematically examined if this ability extends to the detection of non-humanoids' contingent actions in a communicative context. We examined two- to three-year-olds' understanding of contingent actions produced by a non-humanoid robot. The robot either responded contingently to the actions of the participants (contingent condition) or programmatically reproduced the same sequence of actions to another participant (non-contingent condition). The results revealed that the participants exhibited different patterns of response depending on whether or not the robot responded contingently. It was also found that the participants did not respond positively to the contingent actions of the robot in the earlier periods of the experimental sessions. This might reflect the conflict between the non-humanlike appearance of the robot and its humanlike contingent actions, which presumably led the children to experience the uncanny valley effect.

## Introduction

The detection of behavioral contingency is one of the abilities that play an important role in human interaction from early stages of development. Previous studies of sensitivity to behavioral contingency have shown that even infants can distinguish whether or not others' reactions are contingent on their own actions. One of the experimental procedures used to examine this sensitivity is the still-face paradigm, in which the experimenter facing the infant participant stops contingent interaction, along with facial and vocal signals (e.g., [1]–[5]). Another procedure which enables stricter control of the effect of contingency is called the double-video paradigm. In this procedure, the infant and the experimenter (or mother) interact via video cameras and TV monitors in the contingent condition, while in the non-contingent condition, a video playback of the actions that the experimenter performed in the contingent condition is shown to the infant. Since the pioneering work by Murray and Trevarthen [6], studies based on this paradigm have shown infants' ability to regulate their interaction with others (e.g., [7]–[10]).

Although the double-video paradigm is a powerful and useful tool for assessing infants' reactions to behavioral contingency, it has limitations. First, only humans can serve as stimuli producing contingent actions, since they can react flexibly to the infant's actions. This limitation makes it difficult to separate the effect of contingency from humans' physical appearance or species-specific actions. Meltzoff and colleagues argued that “like-me” detection, which can be regarded as a kind of conspecific recognition, might function as a foundation of social cognition [11]–[13]. Further, Sanefuji, Ohgami, & Hashiya [14], [15] demonstrated that when shown photographs or point-light displays as stimuli, infants show preference for individuals who are similar to them, suggesting a releasing mechanism of the like-me detection. These studies suggest that the effect of behavioral contingency in humans inevitably interrelates with the ability of “like-me” detection. To analyze behavioral contingency further, it is necessary to extract the effect of behavioral contingency from such a confounding factor.

A second limitation is that interaction through TV monitors, rather than face-to-face interaction, is crucial to equate stimuli between the contingent and non-contingent conditions. Despite that infants can discriminate between two- and three-dimensional displays, and they respond more readily to a live adult [8], a video playback is necessary to produce the non-contingent stimulus. The still-face paradigm might be one way to manipulate social contingency in face-to-face interactions between an infant and experimenter. However, it becomes difficult to contrast the infants' responses with their natural interactions, since the movements of the experimenter are temporarily halted in the still-face paradigm. Thus, without TV monitors and video-recordings, it is technically difficult to present a non-contingent condition while controlling the stimulus properties between the two conditions.

One way to overcome these limitations is to use a non-humanoid robot as the interacting partner. This might allow us to test children's detection of contingency in a non-human agent in relatively natural settings. Further, by programmed recording the actions performed by the robot for a participant in the contingent condition can be reproduced exactly for another participant in the non-contingent condition. Previous studies have suggested that infants attribute mental states to non-human objects including robots when the latter appear to interact with a person [16]–[18]. Though there are some observational reports about the effect of behavioral contingency by a robot in group interaction [19]–[21], there have been few studies which systematically compare participants' reactions to contingent vs. non-contingent actions of a robot.

In the present experiment, we used a creature-like robot that interacts with people through actions and sounds [22]. We predicted that if infants detect behavioral contingency by the robot, they would interact differently depending on whether or not the robot acted contingently. As the first step in this line of research, we tested 2- to 3-year-old children, since children at this age are expected to demonstrate a sufficient level of mobility and a varied social repertoire.

## Methods

### Stimuli

**Figure 1:**
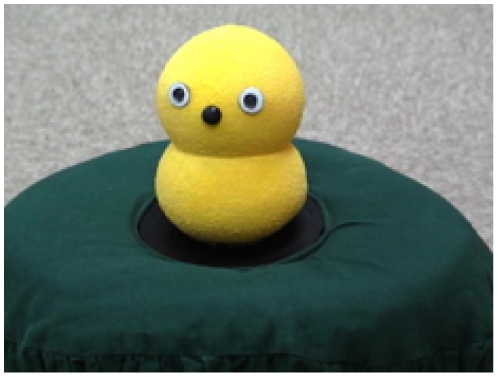
Keepon, the robot used in the experiment.

As the target stimulus for the experiment, we used a robot named Keepon, which was built to study the development of communication [20]–[22] (see Figure 1). Keepon is a small creature-like robot (12 cm in height, 8 cm in diameter) made of silicone rubber and designed such that children can safely interact with it. By coordinating the movement of its four motors, Keepon can turn its face toward a certain target and produce the following actions: nodding (±40 degrees), shaking (±180 degrees), rocking sideways (±25 degrees), and bobbing (15 mm stroke). Two color CCD cameras were incorporated into Keepon's face as eyes, and a microphone was installed as its nose. The operator used the images and acoustic information collected through these instruments to remotely control Keepon's orientation and actions with a computer to which the robot was connected. As control stimuli, 1 stuffed animal, 6 wooden toy blocks, 1 toy ball, and 1 nesting box were also prepared.

### Participants

The participants were 16 children aged 2 to 3 years (*M* = 30.7 months, *SD* = 6.5 months; 8 boys and 8 girls). Their mothers also participated in the experiment. The data for an additional 6 children were excluded from the analysis because of their fussiness during the experiment. All participants were registered on the voluntary panel for infant study at Kyushu University. Written informed consent was obtained from the caretakers of the children before the experiment was conducted. The procedure of the present study complied with the “Ethical Principles of Psychologists and Code of Conduct” (American Psychological Association, 2002).

### Design

To avoid any effect of previous experience in the experiment, we adopted a between-subjects design. The participants were randomly assigned to one of two conditions (4 boys and 4 girls per condition) and tested in one session.

In the contingent condition (C-condition), an experimenter (K.Y.) remotely controlled Keepon through the PC to respond contingently to the actions of the participants and attempted to generate natural interactions as far as possible (see Table 1). The sequence of actions produced by Keepon in the C-condition was recorded by the PC and was replayed in the non-contingent condition (NC-condition).

**Table 1:** List of expressions by Keepon as reactions to participants in the C-condition.

Behavior of participants	Reaction of Keepon
Look at Keepon	Look at participant's eyes
Look at the certain target (mother or toys)	Look at the same one
Show or hold out toys to Keepon	Look at the toy
Indifferent to Keepon	Make a sound
Talk to Keepon/Ask Keepon	Nod (tilt) with (or without) sound
Look intently at Keepon/Ask Keepon	Rock (sideways) with sound
Slap Keepon	Shake (pan) with sound
Touch Keepon	Bob (shrink) with sound

In the NC-condition, Keepon replayed the same sequence of actions as it performed in the C-condition, but now facing a new participant of the same sex and almost the same age (within three months) as in the C-condition. Thus, actions expressed by Keepon were not contingent on the actions of the participant in the NC-condition, but the sequence of actions presented to the 2 participants was exactly the same between the conditions.

### Procedure

**Figure 2:**
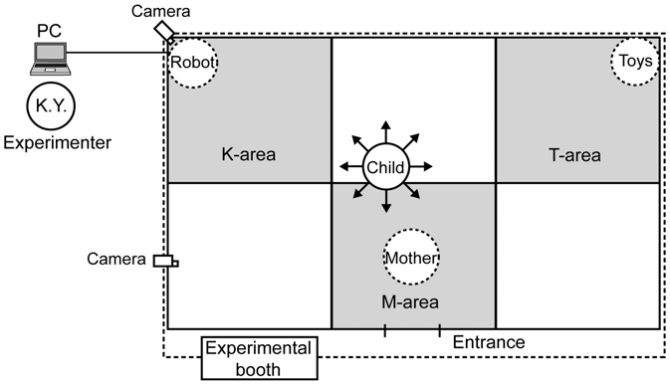
Experimental setting and equipment. Shaded areas represent the areas defined as targets for analysis.

The experiment was conducted in an experimental booth (190×312 cm) built in a room of the university. Figure 2 shows a schematic setup of the experiment. Keepon was kept on one side of the booth (right or left of center, counter-balanced across participants), while the other toys were placed on the opposite side. The experimenters first built rapport with the participant and allowed him or her to familiarize with the environment. The participant then entered the booth with his or her mother. The experiment started upon their entry into the booth and Keepon became active, either under the experimenter's control (C-condition) or programmatically (NC-condition).

During the experiment, the participant was allowed to move and play freely in the experimental booth. On the other hand, the mother was instructed to sit still on the floor midway between Keepon and the toys and to not speak or influence the child, although she was allowed to reply briefly or nod her head in response to questions or comments. The experiment continued until it became apparent that the participant had lost interest in the situation, up to a maximum of 10 minutes.

In addition to Keepon's eye-camera, we installed 2 cameras in the experimental booth in order to complement Keepon's recording of the participant's responses as well as to record the mother's behavior. This was because it became difficult to record the participants' responses solely through Keepon's eye-camera, especially in the NC-condition. Thus, the participants' responses were video recorded from different perspectives through the multiple cameras.

### Analysis

We analyzed the data from the participant's first look at Keepon. Since the minimum duration of a session was 400 sec, the first 400 sec of each video record was set as the target of our analysis. The first and second authors coded each participant's looking behavior and position in the booth on a second-by-second basis. For looking behavior, targets coded were Keepon, mother, or toys. For three randomly selected sessions, 18.8% of the collected data, a new coder blind to the aim of the research independently coded the data. The average inter-coder agreement score was κ = .84, indicating high inter-observer reliability.

To define the participants' position in the booth, we operationally divided the floor of the booth into six spaces and defined the spaces around Keepon, the mother, and the other toys as the K-, M-, and T-areas, respectively. All the spaces were 0.99 m^2^ in area. The locations of all the participants in the booth were coded in the same manner as described above. The concordance rate was .93 on average, indicating high reliability of the coding.

## Results

As the response measures, we took the participants' looking time at Keepon (LTk), the mother (LTm), and the toys (LTt) and the time they spent in the three areas (K-, M-, and T-areas). Specifically, looking time was considered to reflect the participants' interest and the time spent in the three areas indicated the physical proximity of the participants to Keepon, the mother, or the toys.

The statistical analyses consisted of analysis of variance (ANOVA) followed by post-hoc analysis using Ryan's method [23]. Adjusted significance levels (nominal levels) are applied in this method. The nominal level depends upon the number of samples in the group being compared. For all post-hoc analyses we used a significance level of 5%.

### Looking time at Keepon, the mother, and the toys

**Figure 3:**
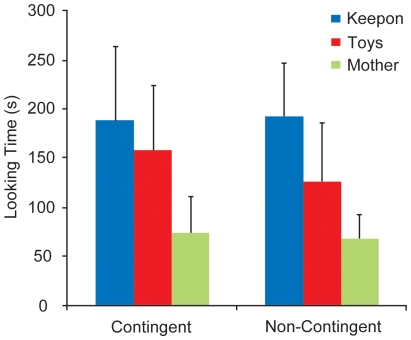
Total looking times at Keepon, the mother, and the toys in the C- and NC-conditions. Vertical bars represent standard deviations.

We compared total LTk, LTm, and LTt in the C- and NC-conditions (Figure 3). A 2 (condition: C and NC) ×3 (target: Keepon, mother, and toys) two-way mixed ANOVA showed a significant main effect of condition (*F* (1, 14) = 4.89, *p* = .044) and target (*F* (2, 28) = 12.58, *p*<.001). The interaction between condition and target was not significant (*F* (2, 28) = 0.30, *p = *.74). A post-hoc analysis demonstrated that LTk and LTt was longer than LTm (LTk > LTm: *t* (28) = 4.99, *p*<.001, nominal level = .017; LTt > LTm: *t* (28) = 2.97, *p* = .006, nominal level = .033).

**Figure 4:**
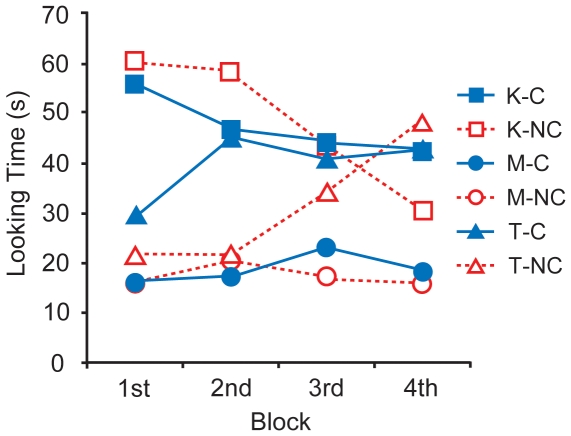
Looking times in the C- and NC-conditions as a function of block.

To investigate how LTk, LTm, and LTt changed with time, we divided the test session (duration of 400 s) into four blocks (100 s per block) and conducted a 2 (condition: C and NC) ×3 (target: Keepon, mother, and toys) ×4 (block: 1^st^, 2^nd^, 3^rd^, and 4^th^) three-way mixed ANOVA. As shown in Figure 4, the results indicated a significant two-way interaction between target and block (*F* (6, 84) = 6.82, *p*<.001) and three-way interaction among the three factors (*F* (6, 84) = 2.60, *p* = .023), along with the significant main effect of target and condition as already described above. Analysis of the three-way interaction (target × condition × block) revealed that LTt in the C-condition was longer than in the NC-condition during the 2^nd^ block (*F* (1, 168) = 7.57, *p* = .007). The simple main effect of block of LTk was significant in the NC-condition (*F* (3, 126) = 11.51, *p*<.001) and the simple main effect of block of LTt was significant in both conditions (C: *F* (3, 126) = 3.07, *p* = .030; NC: *F* (3, 126) = 9.34, *p*<.001). (Since we mainly focused on the difference between the C- and NC-conditions, detailed analysis of the two-way interaction is omitted).

A post-hoc analysis of the simple main effects showed that in the C-condition, LTt during the 1^st^ block was significantly shorter than during the 2^nd^ block (*t* (126) = 2.80, *p* = .006, nominal level = .008) and marginally shorter than during the 3^rd^ block (*t* (126) = 2.01, *p* = .046, nominal level = .025) and the 4^th^ block (*t* (126) = 2.37, *p* = .019, nominal level = .013). On the other hand, in NC-condition, LTt during the 4^th^ block was significantly longer than 1^st^, 2^nd^, and 3^rd^ blocks (4^th^>1^st^: *t* (126) = 4.53, *p*<.001, nominal level = .013; 4^th^>2^nd^: *t* (126) = 4.55, *p*<.001, nominal level = .008; 4^th^>3^rd^: *t* (126) = 2.46, *p* = .015, nominal level = .025), and marginally longer during the 3^rd^ block than during the 1^st^ block (*t* (126) = 2.08, *p* = .040, nominal level = .025). Moreover, in the NC-condition, LTk during the 1^st^ and 2^nd^ blocks were significantly longer than during the 3^rd^ and 4^th^ blocks (1^st^>3^rd^: *t* (126) = 2.90, *p* = .004, nominal level = .013; 1^st^>4^th^: *t* (126) = 5.11, *p*<.001, nominal level = .008; 2^nd^>3^rd^: *t* (126) = 2.59, *p* = .011, nominal level = .025; 2^nd^>4^th^: *t* (126) = 4.79, *p*<.001, nominal level = .013), and marginally longer during the 3^rd^ block than during the 4^th^ block (*t* (126) = 2.20, *p* = .029, nominal level = .025).

### Time spent in the three areas

**Figure 5:**
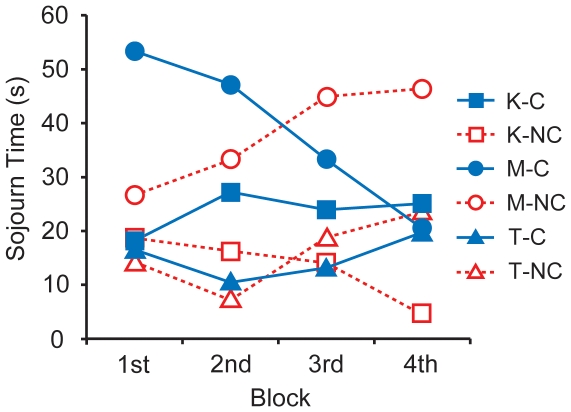
Mean sojourn times in the K-, M-, and T-areas in the C- and NC-conditions as a function of block.

The time spent by each participant in each of the three areas (K-, M-, and T-areas) was used for the analysis. As shown in Figure 5, a 2 (condition: C and NC) ×3 (area: K-, M-, and T-areas) ×4 (block: 1^st^, 2^nd^, 3^rd^, and 4^th^) three-way mixed ANOVA yielded the following: (a) a marginal main effect of area (*F* (2, 28) = 2.82, *p* = .077), (b) a significant interaction between condition and block (*F* (3, 42) = 4.42, *p* = .009), and (c) a three-way interaction among the three factors (*F* (6, 84) = 3.25, *p* = .006). Analysis of the condition × block interaction showed that the time spent in the three areas was longer in the C-condition than in the NC-condition in the 1^st^ block (*F* (1, 56) = 5.76, *p* = .020) and 2^nd^ block (*F* (1, 56) = 5.66, *p* = .021). The simple main effect of block in the C-condition approached significance (*F* (3, 42) = 2.40, *p* = .081).

Analysis of the three-way interaction (condition × area × block) revealed that in the 1^st^ block, the time spent in the M-area was marginally longer in the C-condition than in the NC-condition (*F* (1, 168) = 3.59, *p* = .060), while in the 4^th^ block, it was marginally longer in the NC-condition than in the C-condition (*F* (1, 168) = 3.38, *p* = .068). The simple main effect of area was significant in the 1^st^ block in the C-condition (*F* (2, 112) = 3.14, *p* = .047), and significant in the 4^th^ block in the NC-condition (*F* (2, 112) = 3.20, *p* = .044). In addition, the simple main effect of block in the M-area was significant in both conditions (C: *F* (3, 126) = 6.73, *p*<.001; NC: *F* (3, 126) = 2.82, *p* = .042).

A post-hoc analysis of the simple main effects demonstrated that in the C-condition, children tended to spend more time in the M-area than in the T- and K-areas during the 1^st^ block (M > T: *t* (112) = 2.22, *p* = .029, nominal level = .017; M > K: *t* (112) = 2.12, *p* = .036, nominal level = .033), while in the NC-condition, they spent more time in the M-area than in the K-area during the 4^th^ block (*t* (112) = 2.53, *p* = .013, nominal level = .017). Moreover, in the C-condition, the time spent in the M-area was longer during the 1^st^ and 2^nd^ blocks than during the 4^th^ block (1^st^>4^th^: *t* (126) = 4.10, *p*<.001, nominal level = .008; 2^nd^>4^th^: *t* (126) = 3.34, *p* = .001, nominal level = .013), and marginally longer during the 1^st^ block than during the 3^rd^ block (*t* (126) = 2.51, *p* = .013, nominal level = .013). In contrast, in the NC-condition, they tended to spend more time in the M-area during the 3^rd^ and 4^th^ blocks than during the 1^st^ block (4^th^>1^st^: *t* (126) = 2.47, *p* = .015, nominal level = .008; 3^rd^>1^st^: *t* (126) = 2.29, *p* = .024, nominal level = .013).

## Discussion

The present study provides evidence that behavioral contingency between the participant and the non-humanoid robot can have a marked effect on the children's reaction. The 2–3 year old children changed their behaviors depending on whether or not the robot's actions were contingent on their own actions.

This might empirically support the view that the children attribute mental states to non-human objects that appear to interact with people, as suggested in younger children or infants [16]–[22]. However, some aspects of our results were inconsistent with a natural prediction that the children would respond more communicatively to Keepon when it behaved contingently: there was no significant difference between the C- and NC-conditions in terms of the overall LTk or overall time spent in the K-area. On the other hand, though LTk decreased with time in the NC-condition, such a tendency was not observed in the C-condition. This suggests that observable features of Keepon, including its physical attractiveness or the way it acts, do not fully explain the present results. Considering that the time spent near to the mother tended to increase in the NC-condition, the decrease in looking time at Keepon in the NC-condition can be interpreted as habituation or fatigue, which is defined as a decline in responsiveness with repetitive stimulations [24]. In contrast, in the C-condition, contingent reactions by Keepon might have maintained the participants' interest.

The significant interaction between the blocks and conditions highlights another interesting aspect of the present results. In the 1^st^ block, there was no significant difference between the C- and NC-conditions in LTk, LTm, or LTt. However, the mean time spent in the M-area in the 1^st^ block was marginally higher in the C-condition than in the NC-condition. In addition, in the C-condition, the time spent in the M-area tended to be higher than in the other two areas in the 1^st^ block, whereas no such tendency was found in the NC-condition. These differences indicate that the area used by the participants was limited to the space around their mothers during the 1^st^ block in the C-condition; however, in the NC-condition, they moved around more freely. In other words, the participant stayed by the mother and restricted the chance to approach to Keepon with keeping attention to it at the earlier stages of the C-condition.

Such a response can be reasonably interpreted as reflecting the participants' hesitation to interact with Keepon when they experienced it reacting contingently. We consider the possibility that this response pattern could be related to the uncanny valley hypothesis [25]. This is a theoretical hypothesis to describe the relationship between the human-likeness of a robot and the sense of its familiarity perceived by humans. As a robot's physical appearance (in combination of its movement) becomes more humanlike, the sense of familiarity with the robot generally increases. However, at a point where its appearance becomes quite similar to humans, people begin to perceive it as uncanny and unfamiliar, although such a tendency diminishes when they can no longer find any perceivable difference. This tendency of human perception might arise from their ability to recognize human-likeness or conspecific agents [14], although the mechanism underlying such perception is still under discussion [26].

Animals including humans use multiple signals to accurately detect conspecifics [27]–[30]. Considering behavioral contingency as one of the signals for human-likeness, it should be reasonable to expand the range of application of the uncanny valley hypothesis from the domain of physical appearance to the other domains relevant to human-likeness or to combinations of domains. The present study supports this view in that the odd combination of humanlike (contingent) movements and non-humanlike appearance of a robot induced hesitation by the participants.

Though the present study tested 2- to 3-year-old children, previous studies have demonstrated that even 2-month-old infants can detect social contingency in adult-infant interactions and tend to show positive responses to it [9]. Adults or older children in industrialized countries might rapidly overcome an uncanny valley effect by applying the concept of “robot” to Keepon. However, for individuals who perceive human-likeness with only limited or no knowledge of the robot, it should be adaptive to require a period of habituation and learning before approaching or exploring a non-humanoid that moves contingently. This might be the strategy used by the 2–3-year-old children in the present study.

The results also suggest that children who interacted with Keepon in the C-condition overcame the uncanny valley with experience: though the looking time at Keepon, mother, and toys remained almost stable over the session (except that LTt in the 1^st^ block was lower than other 3 blocks), the time spent near to the mother decreased as the session proceeded. A previous study suggested that 60 sec of observation altered 10-month-old infants' perception of agency of a humanoid robot [16]. According to their looking time, the infants seemed to regard the robot as a communicative agent, though they regarded it as an object in the non-interactive or non-active conditions. Those results are broadly consistent with the present findings in suggesting a role of experience in the detection of agency.

However, we cannot conclude on the basis of our results that the participants began to interact communicatively with Keepon during the 400 sec experimental session. Conceivably, the discrepancy between the non-humanlike appearance of Keepon and its humanlike contingent actions was too large for the children to integrate within the limited time span. Another possibility should be the gap between the detection of agency and engagement through overtly communicative interaction with the agent in face-to-face situations. These possibilities remain to be addressed in future studies aimed at clarifying the basis for human-robot interaction in more detail. In addition, relevant experience with nonhuman agents before the age of 2 years, such as toys, might have influenced the present results. Since strict control of the developmental history of children is not practical, a cross-cultural study would be one way to reveal the effect of experience on the perception of human-likeness.

Presenting various robots that differ in appearance and performance would open a new direction of research to address empirically what forms the basis of human interaction, as Keepon functioned as a platform to shed light on a new aspect of the uncanny valley effect and as a valuable mirror to reflect humans' perception of themselves.
